# Real-time analysis of soccer ball–player interactions using graph convolutional networks for enhanced game insights

**DOI:** 10.1038/s41598-025-05462-7

**Published:** 2025-07-01

**Authors:** Fahad Majeed, Maria Nazir, Kamilla Swart, Marco Agus, Jens Schneider

**Affiliations:** 1 College of Science and Engineering, Hamad Bin Khalifa University, Doha, Qatar; 2 Department of Computer Science and AI, CUQ Ulster University, Lusail, Qatar

**Keywords:** Sequential fusion, Soccer player detection, Tracking, Soccer video analytics, Speed estimation, Graph convolutional networks, Engineering, Mathematics and computing

## Abstract

We present a sequential fusion-based real-time soccer video analytics approach designed to comprehensively understand ball–player interactions. Our approach leverages the power of deep computer vision models, employing a CSPDarknet53 backbone for detection and a Graph Convolutional Network (GCN) for predictive analytics. The proposed approach intricately analyzes ball–player interactions by evaluating metrics such as inter-player distances, proximity to the ball, and hierarchical sorting based on shortest distances to the ball. We also track and estimate each player’s total distance and speed covered throughout the game. Our method performs exceptionally well on both uni- and multi-directional player movements, uncovering unique patterns in soccer videos. Extensive experimental evaluations demonstrate the effectiveness of our approach, achieving 91% object detection accuracy, 90% tracking and action recognition accuracy, and 92% speed analysis accuracy on benchmark datasets. Furthermore, our approach outperforms existing GCN techniques, achieving accuracies of 92% in graph connectivity, 89% in node classification, 87% in player tracking, and 88% in event recognition. Here, we show that our method provides a robust and accurate solution for real-time soccer video analytics, offering valuable insights into player performance and team strategies.

## Introduction

The sports industry is a global force, engaging millions of enthusiasts worldwide through a vast spectrum of activities that capture their passion and interest. Soccer, in particular, stands as the most popular sport, with an unparalleled level of fan engagement and viewership^1^. Given the massive popularity of the sport and driven by clubs’ demands, soccer has received massive attention from video analytics. A significant number of sports industries, both commercial and scientific, use videos and images of matches. Identifying and summarizing game events is essential for coaches, sports analysts, and broadcasters^2^. An enabling technology in this context is precise and effective ball–player detection, which is crucial for accurately analyzing interactions and movements on the field. Computer vision-based systems offer non-invasive solutions, as opposed to GPS and other sensors carried by the players on the field. A system that comprehensively addresses the aforementioned issues would provide the opportunity to analyze games holistically, including *quantitatively* assessing the physical and team play capabilities of individual players^3,4^, thus offering immediate value to coaches. Technological advances, particularly in computer vision, are revolutionizing the sports industry by fundamentally altering game analysis, athlete training, performance analysis, and team strategies^5^. As a result, many video analysis approaches have been developed to assist spectators, referees, coaches, and players. However, applications like player/ball detection and tracking, event detection, and game analysis remain challenging tasks^6,7^. A precise and effective ball–player interaction solution is pivotal for automating statistical analysis in sports. Despite this potential, determining the ball’s position and interaction between players on the pitch in wide-angle shots of a soccer game poses a significant challenge^8^. The ball presents a significant challenge due to its small size, partial occlusions, poor lighting, unpredictable trajectories, and size variations caused by camera perspective, especially when compared to other objects on the field and in the surrounding area^9^. The ball’s high speed and poor color separation from players’ white socks and shoes can lead to multiple false positives in the detection process^10^. The understanding of how soccer players’ speed varies according to their proximity to the ball and their specific role on the team as goalkeeper, attacker, midfielder, and defender is still challenging^11^.

Moreover, the physical performance of players still lacks thorough scrutiny within the research domain, presenting a significant challenge in acquiring metrics pertaining to the physical capabilities of soccer players^12^. Although equipping players and the ball with sensors and performing measurements can provide valuable analytics, the challenge of acquiring comprehensive quantitative data on the physical performance of every team in a competition highlights the limitations of current methods and underscores the difficulty of achieving complete performance data^13^. Gathering individual statistical data for each player, including the total distance a player covers throughout the game and their distribution across different speed categories, is essential^4^. Most analyses and evaluations of player performance lack substantial information regarding the physical aspect of the game. Addressing these challenges requires a combination of custom algorithms and techniques.

Graph Neural Networks (GNNs) have emerged as a powerful tool in sports analytics, particularly for modeling complex relationships and interactions in data. GNNs are designed to operate on graph structures, where nodes represent entities, such as players or teams, and edges represent relationships or interactions between these entities^14^. The emergence of deep GNNs and Graph Convolutional Networks (GCNs)^15^ has expanded their applications, especially in modeling spatial arrangements using graphs. In soccer, GNNs and GCNs have been applied to various tasks, including analyzing successful counterattacks^16^, event detection^17^, and predicting defensive play^18^. Despite these advancements, the individual assessment of players in specific game situations remains under-explored in the literature. Evaluating the performance of individual players in team sports requires a combination of custom algorithms and techniques. This includes traditional deep computer vision methods for extracting players’ 3D positions on the pitch and models such as deep GNNs or GCNs to analyze and understand the current match situation.

From a technical perspective, GNNs/GCNs offer advantages going beyond the capabilities of traditional convolutional neural networks (CNNs). Firstly, they eliminate the need to order features in a specific sequence, allowing them to handle a variable number of players in the camera’s field of view. Secondly, they effectively predict missing nodes in the graph, giving them the ability to complete missing tracks for both the ball and the players. Additionally, GNNs enable the direct learning of local and high-level features from the tracking data. The ongoing research on the evolution of machine learning on graphs applied to real-time scenarios marks a significant leap towards validating its efficiency^19,20^. Given the rapid advancement in this field, it becomes imperative to assess the performance of existing GNN architectures against large datasets under consistent experimental conditions. When collecting individual player and team statistics from soccer footage, tracking objects is crucial for determining the overall distance run, ball possession time, or team configuration.

**Fig. 1:**
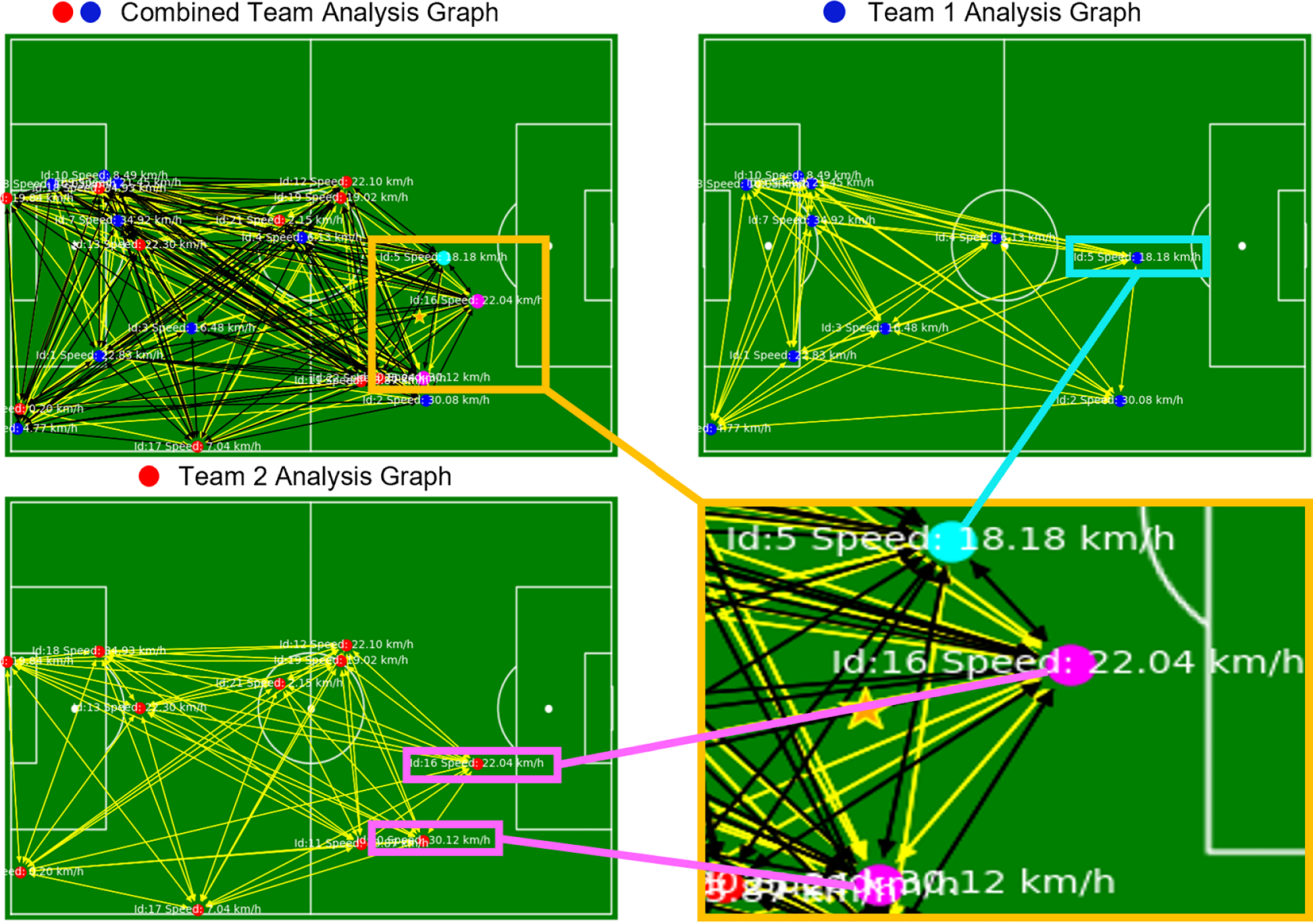
GameFlow visualization. An in-depth analysis of player positions, movements, and interactions during the game. Top Left: A connectivity graph representing both teams, illustrating player IDs, movement speeds, and inter-player connections. Top Right: Player positions, IDs, and speeds for Team 1 (). Bottom Left: Player positions, IDs, and speeds for Team 2 (). Bottom Right: Highlighted players in possession of the ball (magenta  for Team 2, cyan  for Team 1), with the ball represented by an orange .

Considering the challenges mentioned above associated with sports video analytics and the rising popularity of GNNs, this work focuses on a real-time ball–player interaction analysis based on spatio-temporal data using GCNs (see Fig. 1). The proposed approach first employs YOLOv8^21^ to detect players, referees, and the ball in each frame and then links these detections across frames to generate accurate, identity-preserving trajectories in real time. Based on these trajectories, dynamic interaction graphs are constructed for each frame, modeling spatial configurations, motion patterns, and role-based relations among entities. Finally, GCNs are applied to the sequence of graphs to infer tactical behaviors, player movement patterns, and ball interactions, thereby converting low-level spatio-temporal data into high-level, interpretable insights suitable for real-time analysis. The main contributions of this work are as follows:

1. We propose **GameFlow**, a sequential fusion **approach** that decouples the detection model from the GCNs for real-time analysis.
2. We introduce **Soccer++**, a novel dataset containing 17 full soccer games recorded at 1280p resolution and 30FPS, with annotations for three key object classes: player, referee, and ball.
3. We present a novel **method** for accurately tracking and estimating player speed throughout the game, even in complex multi-directional scenarios.
4. Our **approach** effectively manages adaptable spatio-temporal layers, addressing challenges such as player overlap and intricate interactions between players and the ball.
5. We outline **design principles** for sports video analytics to develop robust recommendation systems that enhance the precision and reliability of insights from video data.

To the best of our knowledge, our work is the first to focus on estimating soccer player speed in real-time via sequential fusion in dynamic environments, marking a significant leap beyond conventional methods targeted at rigid objects, such as those used for vehicle speed estimation. While vehicle speed estimation has been extensively studied in the field of transportation and automotive engineering, the estimation of soccer player speed involves unique challenges due to the dynamic and unpredictable nature of sports environments. However, there are parallels between the two domains in terms of the underlying principles of motion estimation, such as analyzing sequential data to infer velocity changes over time. By adapting and extending methodologies from vehicle speed estimation to the domain of sports analytics, our research contributes to bridging the gap between these disparate fields, offering insights and techniques that can potentially benefit both domains.

## Related work

This section reviews the related work in detection, tracking, and analysis using GCNs. We focus foremost on deep learning-based computer vision techniques and GNNs/GCNs in the context of soccer analytics. For a more exhaustive review including classical machine learning methods, we refer the reader to the survey by Akan and Varlı^6^. The advantages and disadvantages of each technique have been highlighted, including several open challenges that need to be addressed before successfully meeting the requirements of soccer video analytics for fully automated recommendation systems.

### Soccer analytics

Multi-object detection is a highly regarded area within computer vision, attracting

significant attention due to its applicability across various fields, including autonomous driving, medical image analysis, object tracking, and robotics. Jiang et al. and Wang et al.^22,23^ provided a solution that seamlessly integrates efficient training tools with the proposed architecture and the compound scaling method. Several studies have proposed visual analytics systems for different domains, each leveraging advanced technologies to enhance decision-making processes. One such system analyzed deep reinforcement learning models for track reconstruction of charged particles in particle physics^24–26^. In a different context, another visual analytics system, GreenSea by Sheng et al.^27^, combined advanced visualization with BLS-based models for tactical analysis, player performance evaluation, and training in soccer. The optimization of the training setup and object detection is achieved by proposing adaptable and efficient training tools, leading to the characterization of their optimized method as a “trainable bag-of-freebies”. Hurault et al.^28^ presented a self-supervised pipeline capable of detecting and tracking low-resolution soccer players under varying recording conditions, eliminating the necessity for ground-truth data. Naik and Hashmi^29,30^ introduced an approach to soccer ball and player tracking based on You Only Look Once (YOLOv3) and Simple Online Real-Time (SORT), aiming to accurately classify detected objects in soccer videos and track them across diverse, challenging scenarios. Maksai et al.^31^ presented a generic and principled method for modeling ball–player interactions, incorporating physical constraints on the ball’s trajectory. A video analysis pipeline is presented by Hongyu et al.^32^ for soccer broadcast footage, incorporating field localization, efficient player tracking, and high-precision ball detection through camera calibration and lightweight models. Nergård et al.^33^ presented an algorithm that detects and annotates the segments of the input data set automatically. The method detects events using sliding windows and categorizes them into a predetermined number of groups. Leo et al.^34^ introduced a real-time multi-view system capable of comprehending interactions between players and the ball. Pappalardo et al.^35^ released an additional dataset featuring spatiotemporal event annotations, emphasizing player statistical analysis.

**Fig. 2:**
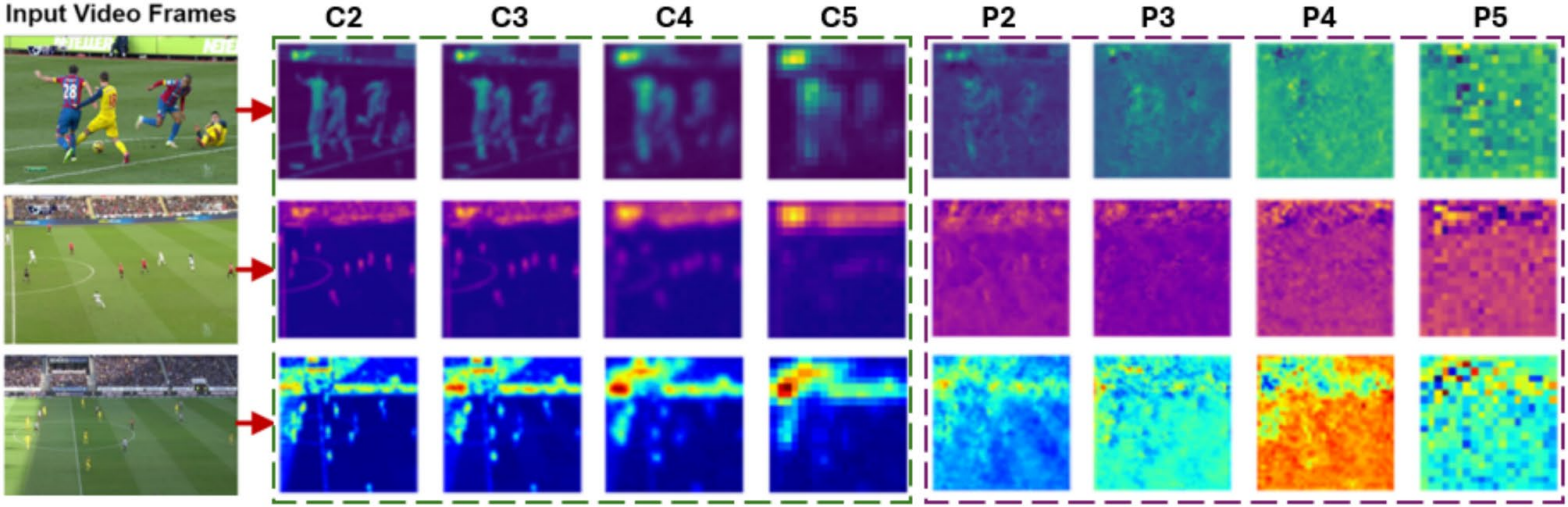
Visualization of backbone and pyramid feature maps from input video frames. Left: Input video frames processed through the Feature Pyramid Network (FPN) to produce feature maps. Right: Backbone feature maps $(C_2 \text { to } C_5)$ and pyramid feature maps $(P_2 \text { to } P_5)$ for three video frames, showing the hierarchical representation of objects at various scales.

### Graph convolutional networks

GNNs have become a key component in 2D image understanding tasks like image captioning, VQA, and image retrieval^36^. GCNs generalized the concept of convolution from the Euclidean domain to the graph domain. Rana^37^ presented a systematic approach for detecting the players and the ball in each frame event based on processing tracking data. Velickovic et al.^38^ described a graph attention network architecture that operates on graph-structured data. Bowen et al.^39^ employed a multi-object tracking and graph analysis system, SocialVis, to monitor social interactions and crowd dynamics in real-time and offline settings. Vaswani et al.^40^ provided a network architecture based solely on attention mechanisms; this transformer-based model eliminates the need for recurrent and convolutional layers. Xie et al.^41^ introduced a broad attentive graph fusion network (BaGFN), a flexible and explicit framework that leverages attentive graph fusion and broad attention modules to effectively capture complex high-order feature interactions in multi-field sparse data. Levie et al.^42^ introduced a novel convolutional architecture which operates in the spectral domain. It is specifically designed for deep learning tasks on graphs. Extending the capabilities of the conventional GCN, a generative adversarial framework is devised by Yu et al.^43^ to enhance the realism of forecasted traffic states, accounting for the joint probabilistic density of actual traffic conditions. An Adaptive Graph Convolutional Network (A-GCN) is introduced to address tactic recognition in sports videos and temporal modeling by Kong et al.^44^ developed an Attentive Temporal Convolutional Network (A-TCN). This approach captures individual and shared data patterns using local and global graphs, facilitating the learning of diverse player interactions. She et al.^45^ introduced an interactive sports visualization system that combines dynamic hypergraphs and narrative visualization with a learning-based SRR-voting model to predict all-star players. Goka et al.^46^ leveraged the spatial-temporal relations among players and prediction uncertainty to enhance the accuracy and robustness of shoot event prediction. The architecture employs a GCNs to capture the spatial connections among team members while utilizing Gated Recurrent Units to analyze dynamic motion data^47^.

### Speed measurement

For the 3D bounding boxes of vehicles along with their tracking and speed estimation, Kocur^48^ leveraged the established geometry of vanishing points within the scene, applying the perspective transformation^49–51^. Sochor et al.^52^ emphasized the calibration of traffic cameras and visual speed measurement using a single monocular camera. Liebe et al.^53^ proposed the FARSEC, an automatic prediction framework for the length of road segments. Their approach uses depth mapping and is capable of managing real-world scenarios, including camera movements and varied video stream inputs. While most research concentrates on estimating vehicle speed, our paper breaks new ground by focusing on real-time soccer player speed estimation. Unlike existing surveys limited to vehicle speed, we introduce a new approach based on sequential fusion patterns. Our contribution lies in connecting sports analytics with computer vision, providing real-time speed assessment and insights into player movements and strategies.

## Methodology

### Architecture

The proposed sequential fusion architecture combines two different models: an object detection model^21^ for training and validation purposes, and a GCN model for refined predictive analysis. The detection model consists of three components: a backbone, a path aggregation network (PANet), and a head. Initially, we give a sequence of video frames of resolution $(608\times 608\times 3)$ as input, denoted as $(i_1,i_2,\ldots ,i_N)$, where each input of video frame $f_i$ is represented as a tensor in $\mathbb {R}^{H\times W\times n}$. Let’s denote the outputs of the object detection model as $\mathcal {O}$ and the outputs of the GCN model as $\mathscr {G}$. The fusion function *F* could combine these outputs to produce a refined output $R_\textrm{fusion}$ as follows:

1 $$\begin{aligned} R_\textrm{fusion}&= (\mathcal {O}, \mathscr {G}) \end{aligned}$$

#### Backbone

In the backbone stage, we extracted the relevant and meaningful features from the input video frames by capturing simple patterns in the initial layers. The proposed approach backbone architecture consists of (CSPDarkNet)^54^ with 53 convolutional layers and integrates cross-stage-partial connections to enhance the flow of information between different layers. We employed a series of downsampling operations using convolutional and pooling layers to extract relevant features, enabling multi-scale feature capture. The input frame then underwent a detection process, where it is divided into smaller segments, reducing the image dimensions to $(896\times 896)$, $(448\times 448)$, $(224\times 224)$, and $(112\times 112)$ before being fed into the convolutional network.

**Fig. 3:**
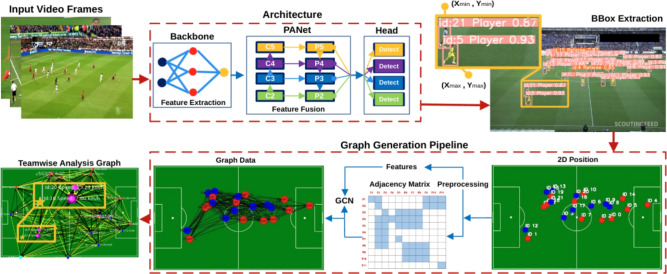
GameFlow architecture. The framework consists of two main components: object detection (top row) and graph-based team analysis (bottom row). Top Row: Object Detection involves a **Backbone** for feature extraction, **PANet** for refining feature resolution, and a **Head** for final object predictions. Bottom Row: GCN processes the detected objects by converting bounding boxes into 2D positions, computing centroids, and constructing an adjacency matrix with relevant features. The resulting graph data is then fed into a GCN for comprehensive team analysis.

#### Neck

The extracted features were then forwarded to the Neck stage for feature fusion, specifically to the Path Aggregation Network (PANet). We used Feature Pyramid Networks (FPN)^55^ to construct a multi-level feature pyramid that enabled the accurate representation of objects at varying scales. The FPN architecture processed a single-scale input image *I* to generate feature maps at different levels. The bottom-up pathway first extracts backbone feature maps $(C_2, C_3, C_4, C_5)$ from conv2, conv3, conv4, and conv5 layers, with strides $\{4,8,16,32\}$ relative to the input image, progressively capturing features at increasing levels of abstraction (see Fig. 2). In the top-down pathway, these backbone feature maps are combined with upsampled feature maps to generate the pyramid levels $(P_2, P_3, P_4, P_5)$. The feature merging process is represented by:

2 $$\begin{aligned} P_i&= \text {Conv}\left( C_i + \text {Upsample}(P_{i+1})\right) \end{aligned}$$

where $P_i$ denotes the final feature map at level $i$, combining high-resolution details $C_i$ with semantic information from $P_{i+1}$. FPN utilized top-down pathways to refine these feature maps through lateral connections, ensuring that each level of the pyramid retains both fine-grained and high-semantic information. This hierarchical structure facilitates the effective detection of objects across multiple scales.

#### Head

The head stage is responsible for making predictions. We achieved this by employing the 4 detection heads of dimensions from $(152\times 152\times 256\times w)$, $(76\times 76\times 256\times w)$, $(38\times 38\times 512\times w)$ and $(19\times 19\times 512\times w\times r)$ that predicted bounding boxes, objectness scores, and class probabilities for each grid cell in the feature map. Afterwards, these predictions were aggregated to derive the final detections. The step-by-step working pipeline of our approach is illustrated in Fig. 3.

### Object localization

After training the object detection $\mathcal {O}$ model, the next step involved applying it to a test dataset, extracting bounding boxes from the predictions to precisely localize and characterize each object of interest. We traversed each video frame and applied the model to recognize objects within the frame. Let $b_i$ represents the $i^{th}$ bounding box in a given frame, defined by the tuple $(x_{min}, y_{min}, x_{max}, y_{max})$, where $(x_{min}, y_{min})$, denote the coordinates of the top-left corner and $(x_{max}, y_{max})$ signifies the coordinates of the bottom-right corner.

3 $$\begin{aligned} b_i = (x_{{\min}}, y_{{\min}}, x_{{\max}}, y_{{\max}}, K_i) \end{aligned}$$

$K_i$ denotes the vector of values associated with the $i^{th}$ bounding box, encompassing pertinent attributes such as object class probabilities or confidence scores. These bounding boxes, along with their associated attributes, are then stored in a structured format, such as a JSON file, for further analysis.

### Graph generation

#### Preprocessing

The process began by converting the extracted bounding box coordinates into normalized 2D positions. To do so, the centroid of each bounding box was divided by the frame’s width and height to result in a unified $[0,1]^2$ coordinate. This allowed us to process frames in different resolutions. In Fig. 3 top-right corner, the bounding boxes are predicted by the $\mathcal {O}$-model, providing pixel coordinates for each bounding box. This 2D positional data is essential for further analysis, such as tracking object movements over time or mapping object interactions within the scene. Additionally, these coordinates are used to generate graph data, providing a visual and analytical representation of the object positions on the soccer pitch. After we have extracted normalized bounding box coordinates, we proceeded with player identification. In this step, players were assigned unique IDs based on their positions and associated with their respective teams.

The next step was feature extraction to capture essential attributes for each object, such as size $(S_i)$, shape $(\phi _i)$, and class information $(C_i)$. These features, along with the bounding box coordinates $(b_i)$, form the node features for the graph. The final node features are concatenated with the adjacency matrix to create the input representation $\varvec{\psi }$ for the GCN. Features extracted from the positional data include normalized coordinates for each player and the ball, and categorical class information (e.g., “player”, “ball”, “referee”).

The resulting input representation $\varvec{\lambda }$ is an $n \times d$ matrix, where *n* represents the number of detected players and *d* denotes the dimensionality of the node features. Given that the number of detected players per frame may vary, this results in variable sizes of the input matrix $\varvec{\lambda }$. To manage this variability, our approach dynamically adjusts the size of the input matrix for each frame, ensuring that the GCN can effectively process the varying number of nodes (players). Specifically, zero padding is employed to standardize the matrix dimensions when necessary. Let $n_t$ denote the number of detected players in frame *t*. The input matrix for frame *t* is initially of size $n_t \times d$. To ensure compatibility with the GCN, the matrix is padded with zero vectors to form a standardized matrix $\tilde{\varvec{\lambda }}_t$ of size $N_{{\max}} \times d$, where $N_{{\max}}$ is the maximum number of nodes considered across all frames. This standardization allows the GCN to handle different *n* values across frames without loss of information. This ensures that the sequential refinement process formalized as:

4 $$\begin{aligned} O_{\mathcal {G}}^{(t)} = \mathcal {G}\left( O_{{DET}}^{(t-1)}\right) , \end{aligned}$$

can be consistently applied regardless of the number of detected players. Here, $O_{{DET}}^{(t-1)}$ serves as the input to the $\mathcal {G}$ at iteration *t*, refining the detection results by incorporating contextual information and spatial relationships. The output $O_{\mathcal {G}}^{(t)}$ represents the refined object predictions after *t* iterations of sequential processing.

### Camera calibration

To accurately process player and ball interactions on the soccer field, we calibrate the camera to map 2*D* image coordinates to 3*D* world coordinates. This calibration involved defining key landmarks on the soccer pitch, such as corners, penalty spots, and the centre circle, as well as dynamic objects like players and the ball. These keypoints are used to establish a reference mapping between the 2*D* image coordinates and the 3*D* field coordinate system. The intrinsic parameters, including focal lengths $(f_x, f_y)$, skew coefficient $s$, and principal point coordinates $(c_x, c_y)$, are estimated using the cv2.calibrateCamera function^56^, which returns the camera matrix encapsulating all these values. Extrinsic parameters, including rotation ($\textrm{R}$) and translation ($\textrm{T}$) vectors, are estimated using cv2.solvePnP^57^ to align the camera’s coordinate system with the soccer field. The transformation from world coordinates to camera coordinates is described by $\textrm{X}_{\text {camera}} = \textrm{R} \textrm{X}_{\text {world}} + \textrm{T}$. To handle the variability in player and ball positions, calibration parameters were integrated with the dynamic tracking system, ensuring accurate conversion of 2*D* image coordinates to 3*D* world coordinates. This allows for the reliable computation of player and ball speeds and distances, where speed is calculated as $\text {Speed} = \frac{\Vert \textrm{X}_{\text {current}} - \textrm{X}_{\text {previous}}\Vert }{\Delta t}$ with $\textrm{X}_{\text {current}}$ and $\textrm{X}_{\text {previous}}$ representing the 3D positions at current and previous frames, respectively, and $\Delta t$ the time interval between frames. Maximum speeds are analyzed by tracking movement trajectories, while team classification is handled separately, relying on the accurate spatial data provided by calibration. Finally, lens distortion is corrected, and reprojection error is minimized. Camera parameters are saved in JSON format for each frame, supporting precise object localization and tracking throughout the game.

#### Graph data

Our graph, $G =(V, E,\varvec{\lambda })$ consists of vertices or nodes for each of the

players, the ball, and the two goals. We dynamically inserted or removed edges between these nodes based on our analysis of the game. In addition, we stored node annotations $\varvec{\lambda }$. Internally, our graph is represented by its adjacency matrix $\varvec{\psi }$. Edges are inserted based on proximity, measured in Euclidean distance, using a cutoff threshold parameter $\delta$. If two players are closer than $\delta$ from each other, we insert an edge to indicate a potential interaction. An interesting aspect of this graph is the bipartite structures arising from players of different teams and non-bipartite structures from player interactions of the same team.

**Fig. 4:**
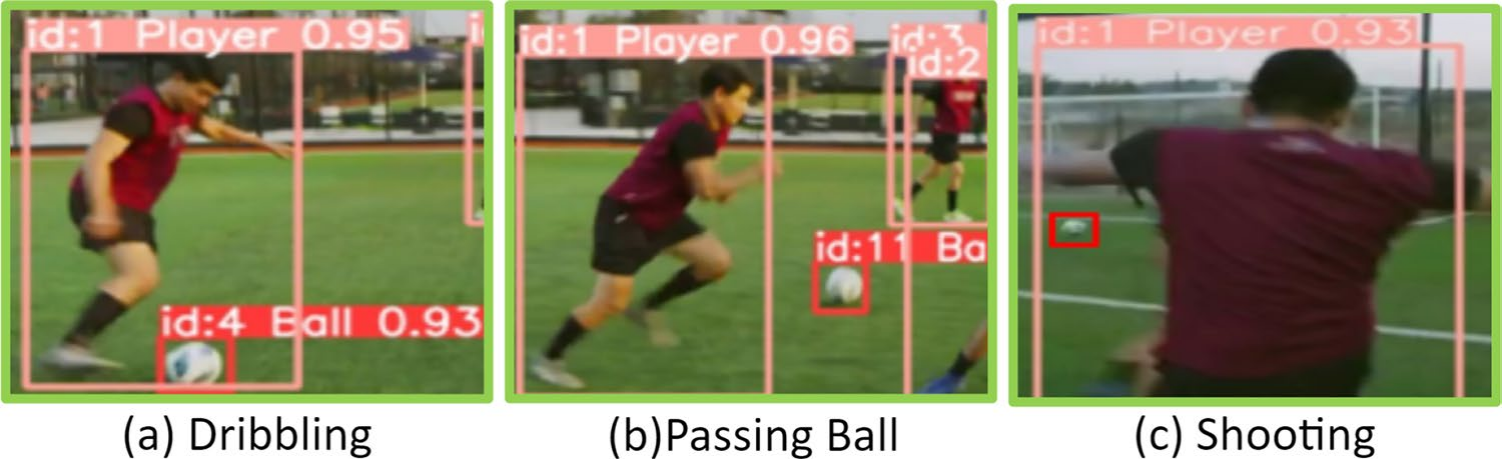
Node and edge construction in soccer. This figure shows how nodes and edges are formed in different game situations, such as dribbling (player-to-ball), passing (player-to-player), and shooting (player-to-goal), along with other possible connections.

Our node labels consist of both positional coordinates $(x_i, y_i)$ and a corresponding class for each of the $n = |V|$ nodes, collectively represented as $\varvec{\lambda }$; combined with the feature matrix $\varvec{\psi }$, they serve as input to the GCN model for refined predictive analysis. The graph data encapsulates the spatial and interaction dynamics of the game, allowing for advanced analysis and predictive modeling using the GCN framework. In the proposed approach, the construction of nodes and edges covers a wide range of soccer scenarios, including but not limited to the following examples.

***Player to player edges:*** When players from the same team are in close proximity or when one player passes the ball to another, an edge is created between their nodes to represent their spatial or tactical connection.

***Player to goal edges:*** An edge is formed between a player node and the goal node when a player attempts a shot, capturing the offensive action towards scoring.

***Player to ball edges:*** Dribbling actions are captured by creating an edge between the player node and the ball node, representing the player’s control over the ball (see Fig. 4).

**Fig. 5:**
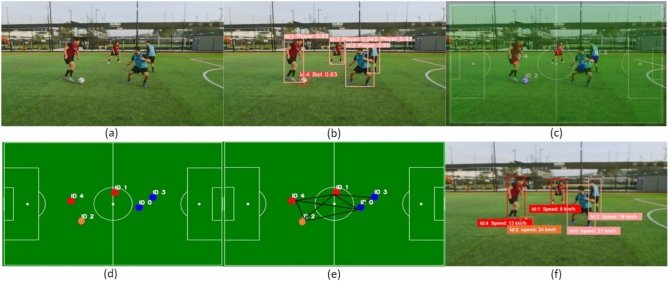
Graph generation pipeline. This figure illustrates the step-by-step process of transforming a video frame into graph data for analysis: (**a**) Input video frame, (**b**) Object detection with assigned IDs and confidence scores, (**c**) Radar view showing extracted 2D positions using centroids, (**d**) Mapped 2D positional data on the soccer pitch, (**e**) Constructed graph based on positional data, (**f**) Final processed output.

#### GCN for predictive analysis

To effectively model spatial and contextual relationships among players, we applied a GCN to this graph representation. Fig. 5 shows the sequential flow of our data generation process. The GCN is designed to refine the predictions of player interactions, movement trajectories, and tactical behaviors by leveraging both local neighborhood information and global structural patterns within the graph. The proposed architecture comprises a stack of three Graph Convolutional layers followed by two Fully Connected (FC) layers to capture both relational dynamics and high-level semantic patterns. The GCN layers are responsible for propagating and aggregating features across the graph structure, enabling each node (player/object) to encode contextual information from its neighbors. The FC layers act as nonlinear classifiers / regressors based on these enriched node embeddings. We adopt the standard GCN propagation scheme introduced by Kipf and Welling^58^, where each node’s representation is updated by combining its own features with those of its neighbors via a symmetrically normalized adjacency matrix.

Following the GCN layers, global max pooling is applied to aggregate node features into a graph-level representation when required (e.g., team-level predictions). The two FC layers then act on either individual node outputs (for per-player prediction tasks) or on pooled representations (for team-level outcomes such as formation strength, press intensity, etc.). The GCN model consists of three layers with dimensions $d \rightarrow 64 \rightarrow 128 \rightarrow 128$, each followed by ReLU activations and dropout (0.3) in the first two layers, followed by two fully connected layers ($128 \rightarrow 64 \rightarrow C$), where *C* denotes the number of prediction classes.

### Speed analysis

#### Source and target ROIs

Initially, the source ROI, designated as $Soccer_{\text {pitch}}$, is constituted by a set of vertices represented as a $Z \times 2$ numpy array, where *Z* denotes the number of vertices. These vertices denote the corners of the soccer pitch in the original video frame, thus defining the spatial extent of the source region. The representation of the source ROI is shown below:

5 $$\begin{aligned} \mathrm {Soccer_{\text {pitch}}} = \left[ {\begin{smallmatrix} j_1 & k_1 \\ j_2 & k_2 \\ \vdots & \vdots \\ j_Z & k_Z \end{smallmatrix}}\right] . \end{aligned}$$

Here, $j_i, k_i$ are the coordinates of the $i^\textrm{th}$ vertex. Moreover, denoting the target dimensions as $W_\text {target}$ (width) and $H_\text {target}$ (height), we can establish the geometric parameters of the transformed soccer pitch. The target ROI denoted as $T_{\text {ROI}}$, is then formulated as a corresponding $L \times 2$ NumPy array, where *L* represents the number of vertices representing the modified spatial boundaries of the soccer pitch in the transformed frame. The matrix of the target ROI is given below.

6 $$\begin{aligned} \mathrm {T_{\text {ROI}}} = \left[ {\begin{smallmatrix} 0 & 0 \\ W_{\text {target}} - 1 & 0 \\ W_{\text {target}} - 1 & H_{\text {target}} - 1 \\ 0 & H_{\text {target}} - 1 \\ \end{smallmatrix}}\right] . \end{aligned}$$

Here, $(0, 0)$ and $(W_{\text {target}} - 1, H_{\text {target}} - 1)$ are the top-left corner and the bottom-right corners. Table 1 shows the transformation process instantiated by a video frame generator initialized with the source video path. After frame acquisition, a replica frame called $annotated\_frame$, is generated to visualize the transformation. Utilizing the video analysis library function $draw\_polygon$, the source polygon delineated by $S_{polygon}$ is superimposed onto $annotated\_frame$, in a user-defined colour and thickness. Finally, the resultant annotated frame is displayed via the $plot\_image$ function, facilitating visual confirmation of the source ROI delineation on the original video frame. This rigorous mathematical exposition elucidates the meticulous procedure for defining and transforming the ROIs, underpinning the fundamental principles driving the video analysis framework.

**Table 1:** Overview of source and target ROIs data. The source points define the region of interest in the original frame, while the target width and height define dimensions that set the standardized reference plane, ensuring consistent spatial representation for analysis.

Data	Value
Source Points	[[100, 300], [1580, 300],
	[7080, 2500], [-1000, 9000]]
Target Width	100
Target Height	300
Transformed Target	[[0, 0], [199, 0], [199, 499],
	[0, 499]]

#### Perspective transformation

The ViewTransformer class is applied to implement perspective transformation. Given source points $S_{points}$, and target points $T_{points}$, represented as 2D NumPy arrays, it computes the perspective transformation matrix $t_m$ using the getPerspectiveTransform function from OpenCV. The transformation matrix $t_m$ is a $3\times 3$ matrix that maps points from the source to the target space. To apply the transformation, the $transform\_points$ method reshapes input points *P* into a suitable format and then computes the transformed points $P^{\prime }$ using the equation $P^{\prime } = {t_m} \cdot {P}$, where the transformation of each point $P=(c,d)$ to its transformed counterpart $P^{\prime }=(c^{\prime }, d^{\prime })$. The ViewTransformer class ensures numerical precision by casting points to float 32 arrays.

#### Speed estimation

The proposed approach is rooted in kinematic principles and mathematical formalism to characterise the movement dynamics of individual objects within the visual domain. The estimation of an object’s speed, a pivotal aspect of our analysis, is formulated as follows:

Let $v_{speed}$ represents the speed of an object, *t* denotes the time interval between consecutive frames, $\Delta x$ denotes the object’s position change between frames, and $\Delta t$ denotes the time interval between measurements. Utilizing the basic kinematic equation: $v_{speed} = \frac{\Delta x}{\Delta t}$. Where we estimate the object speed ($v_{speed}$) by computing the ratio of the change in position ($\Delta x$) to the time interval ($\Delta t$). To account for variations in frame rate (*f*), field dimensions ($f_{dimension}$), and pixel resolution ($R_{pixel}$), we introduce the following adjustments:

#### Frame rate adjustment: $\Delta t = \frac{1}{f}$

This adjustment normalizes the time interval between frames to account for variations in frame rate, ensuring consistent speed estimation across different video sequences.

#### Spatial adjustment: $\Delta x = \frac{d_p}{d_f} \times \Delta s$

Here, $\Delta s$ represents the observed change in the position of the entity in pixel units, $d_p$ represents the pixel width of the field, and $d_f$ represents the actual width of the field in meters. This adjustment scales the observed change in position to account for differences in pixel resolution and real-world dimensions. Incorporating these adjustments into the speed estimation equation as

7 $$\begin{aligned} v_\text {speed}&= \frac{\frac{d_p}{d_f} \times \Delta s}{\frac{1}{f}},\;{\mathrm {from which follows}}\\ v_\text {speed}&= f \times \frac{d_p}{d_f} \times \Delta s. \end{aligned}$$

Furthermore, to introduce stochasticity and enhance the realism of entity movement, we employ a randomization process based on the principles of probability theory. Specifically, for each detected entity, we randomly select a movement direction (left, right, up, or down) and apply a corresponding adjustment to its position within the frame. This stochastic element introduces variability in entity trajectories, mimicking the unpredictability inherent in real-world scenarios and adding depth to our analysis of sports dynamics. Table 2 shows the speed estimation parameter of a typical player in meters per frame.

**Table 2:** Speed estimation parameters of a soccer player. This includes detection thresholds, frame rate, and calibration values to convert movement from pixels to meters per second for accurate analysis.

Category	Parameter	Value
Model Settings	Confidence Threshold	0.25
	IOU Threshold	0.5
	Model Resolution	1280
Video and Motion	Frame Rate (fps)	30
	Player Speed (m/s)	5
	Movement Step (m/frame)	5/30
Field and Projection	Field Width (m)	68
	Video Width (pixels)	1920
	Pixels per Meter	1920/68
	Movement Step (pixels)	$\left( \frac{5}{30} \right) \times \left( \frac{1920}{68} \right)$

To ensure real-time and precise speed estimation during gameplay, we utilized specific metrics such as the Euclidean distance between consecutive positions $(p_1, q_1)$ and $(p_2, q_2)$. We then divided the distance by the elapsed time and converted it to kilometres per hour, $S_{ED} = \frac{d_{ED}}{t} \times 3.6$. This calculation provides a comprehensive overview of the player’s velocity throughout the game. In addition, we employed the absolute difference method to quantify the speed of soccer players. This method assesses the magnitude of displacement between specific points, such as the player’s initial and final positions, represented as $d_{AD} = |start_{point} - end_{point}|$. It enables the evaluation of instantaneous speed at discrete time points by dividing the absolute difference by the time interval, yielding $S_{AD} = \frac{d_{AD}}{t_{interval}}$. This approach offers insights into the player’s velocity at distinct moments during gameplay, enhancing the granularity of our analysis.

## Experimental setup

### Datasets

We constructed the Soccer++ dataset by extracting frames from soccer match videos through a custom video_to_frame conversion Python script. The extracted frames were annotated using Roboflow’s^59^ platform, which facilitated both the preprocessing and organization of the dataset. This dataset was then utilized for training the object detection $\mathcal {O}$ model, ensuring efficient management and consistency throughout the annotation pipeline. The dataset includes three distinct object classes: Player, Referee, and Ball, each carefully labelled to enhance model accuracy in detecting and differentiating these entities in soccer match scenarios. The training set contains 2,203,200 frames (80%), used for model training. The validation set comprises 275,400 frames (10%), which is used to assess model performance during training and for hyperparameter tuning. Finally, the test set consists of 275,400 frames (10%), used for evaluating the final model performance. Table 3 provides statistics on the annotations of our Soccer++ dataset across all three classes. To enhance the quality and versatility of 2*D* positional data for players and the ball, we employed a subset of the SoccerNet dataset^60^, which is the most extensive public repository of soccer videos, containing 550 full broadcast games from Europe’s six premier soccer championships. This enabled us to conduct more experiments and inferences effectively.

**Table 3:** Annotation statistics of our soccer++ dataset.

Class	Unique Tracklets	Total annotations
Player	3,124	1,701,840
Referee	904	503,280
Ball	768	548,880
Total	4,796	2,754,000

**Fig. 6:**
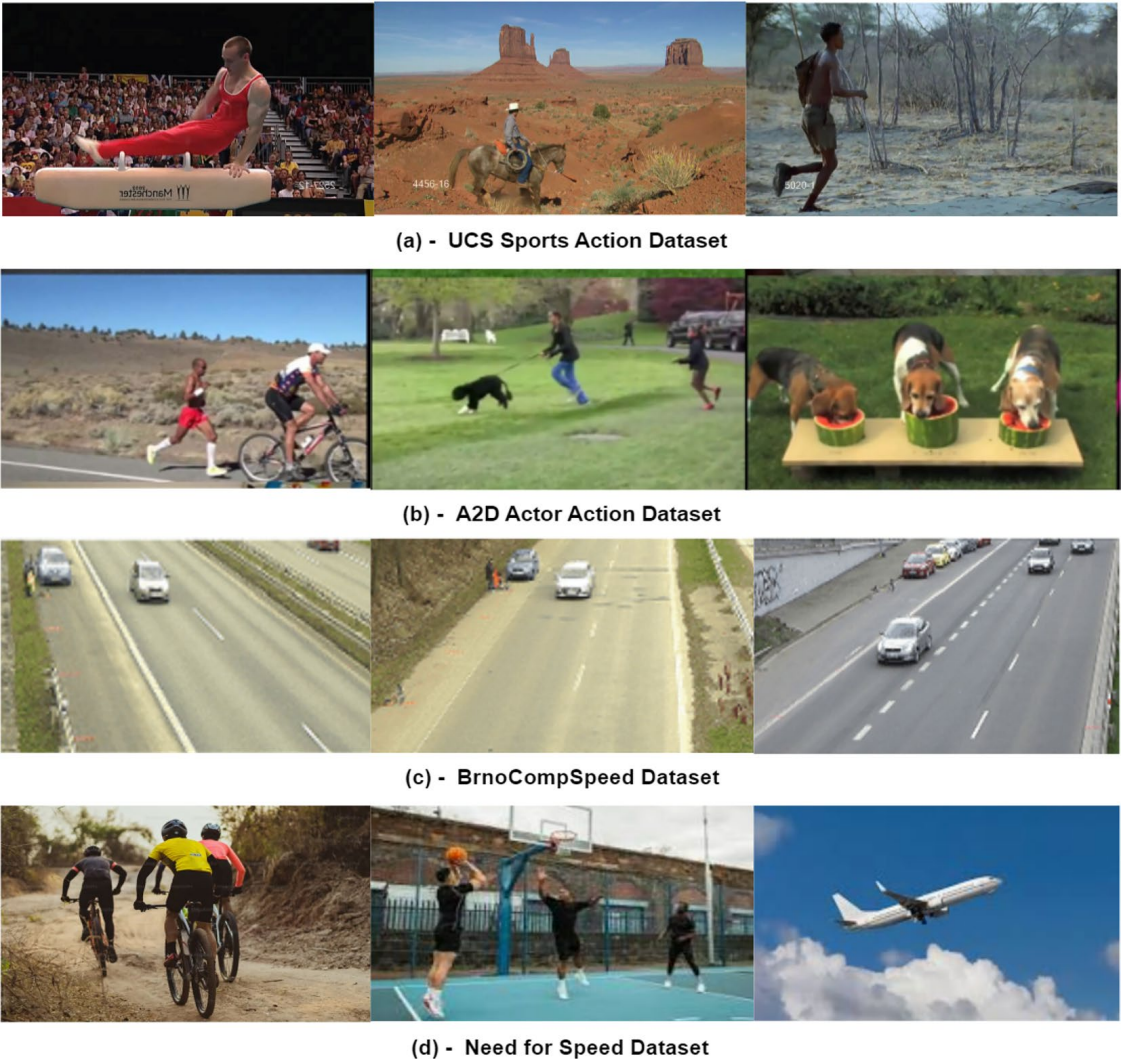
Benchmark datasets: The figure displays sample images from each dataset, showcasing the variety of data they contain.

We also validated our proposed approach on additional benchmark datasets, specifically categorized into Tracking, Action Recognition, and Speed Analysis. The **UCF Sports Action** dataset consists of 137 videos with bounding box annotations, covering 10 distinct human actions^61^. The **A2D Actor Action** dataset includes 3, 782 videos with pixel-level annotations, featuring 9 actors performing 8 different actions, used to model actor-action interactions^62^. For speed analysis, the **Need for Speed (NfS)** dataset provides 100 videos (380K frames) captured at 240 FPS, annotated with bounding boxes and nine visual attributes, including occlusion and fast motion^63^. Lastly, the **BrnoCompSpeed** dataset contains 21 full-HD videos, each approximately 1 hour long, with 20, 865 vehicle instances annotated with precise speed measurements using LiDAR and GPS, aiding in speed analysis and comparison^48^. Table 4 presents the detailed information, while Fig. 6 displays sample images from each of the benchmark datasets.

**Table 4:** The table outlines key details of the benchmark datasets, including the number of videos, annotation types, and class categories.

Dataset	Videos	Annotations	Classes
Tracking and action recognition datasets			
UCF Sports	137	Bounding Boxes	10 Actions
A2D Actor	3782	Pixel level	8 Actions, 9 Actors
Speed analysis datasets			
Need for Speed	100	Bounding Boxes	9 Attributes
BrnoCompSpeed	21	Speed	Vehicles (20,865)

### Models

#### Object detection

The proposed approach leverages an object detection model based on a cross-stage partial network (CSPDarkNet 53) as backbone architecture with feature pyramid network^55,64^ as neck architecture. We evaluated our model on the validation set with $mAP_{(50)}$ and $mAP_{(50-95)}$ after every epoch. If there is no improvement in the mAP for 5 consecutive epochs, we reduce the learning rate by a factor of 10. Furthermore, we used a simple data augmentation process to randomly apply horizontal flipping and color jittering to each training sample. As an early stopping strategy, we cut off the model’s training if no improvement is made concerning the mAP on the validation set for ten consecutive epochs or if the training reaches 300. All the videos have a resolution of 1280*p* and are recorded at 30 FPS. All hyperparameters remained constant during the complete training and validation procedure.

#### GCNs

The object detection output $\mathcal {O}$ is processed into graph-compatible data for the GCN $\mathscr {G}$ model by extracting centroid-based 2D positional features from bounding boxes, enriched with class and motion information. The adjacency matrix $\psi$ is computed based on spatial distances, temporal consistency, and team formations. Nodes incorporate player-specific attributes (e.g., role, speed, direction), while edges capture relative positions and distance-weighted attention scores. NetworkX is used to construct and manage the interaction graphs. The GCN $\mathscr {G}$ performs spatiotemporal analysis to model player-ball interactions, including identifying ball possession, nearest players, movement dynamics, and inter-player distances. This enhances both predictive and classification accuracy at the team level. Visualizations of these metrics (e.g., player distance, speed, ball movement) are generated using Seaborn and Matplotlib. A three-layer feedforward GCN architecture is adopted to balance expressiveness and avoid over-smoothing, as deeper networks degraded performance and shallower ones underfit complex interactions. Regularization techniques include dropout (0.3probability) between layers and optional batch normalization for deeper variants. Skip connections were evaluated but excluded due to marginal impact. Training is conducted end-to-end using cross-entropy loss for interaction classification and mean squared error (MSE) loss for trajectory refinement. The model is trained over 300 epochs with a learning rate of 0.001 using Adam optimizer (weight decay = 1e-5), with batch size 32 and early stopping based on validation accuracy.

### Loss functions

**Fig. 7:**
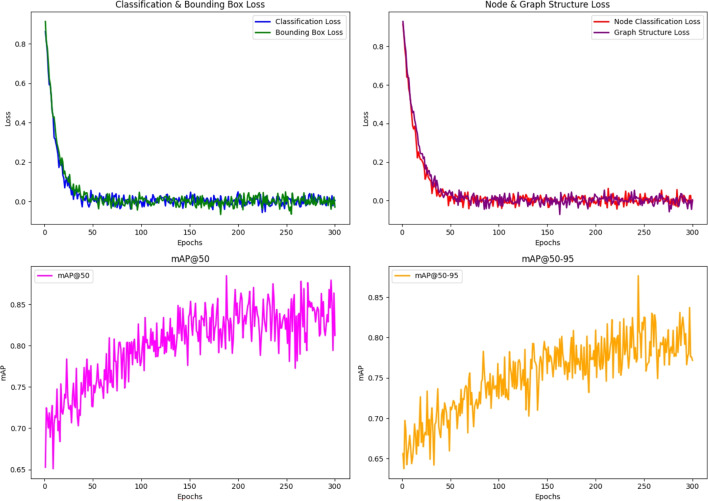
Loss & mAP Curves: The figure displays four key performance metrics across 300 epochs. Top-Left plot shows the loss curves for Classification in blue , Bounding Box in green , while Top-Right plot shows the Node Classification in red , and Graph Structure in purple  losses, highlighting model convergence. The Bottom-Left plot shows the $mAP_{(50)}$ object detection accuracy in magenta  curve, while the Bottom-Right: plot shows the $mAP_{(50-95)}$ overall model performance in precision and recall in orange  curve, with metrics approaching optimal values by the end of training.

The proposed approach utilizes a combination of loss functions tailored to both the object detection $\mathcal {O}$ model and the GCN $\mathscr {G}$ model. Our approach follows a sequential processing pipeline, where the $\mathcal {O}$ model is initially trained independently, as illustrated in the top-left and top-right sections of Fig. 7. The outputs from this model are then fed into the $\mathscr {G}$ model for refined predictive analysis. These loss functions ensure that the system can accurately detect objects (players, ball) and model their interactions effectively within the graph structure.

#### Object detection loss

The object detection model is trained using two primary loss components:

**Classification loss:** The classification loss is used to penalize incorrect predictions of object classes (e.g., players, ball). We use the Cross-Entropy Loss, defined as:

8 $$\begin{aligned} \mathcal {L}_{\text {cls}} = -\sum _{i=1}^{N} \sum _{c=1}^{C} y_{ic} \log \hat{y}_{ic}, \end{aligned}$$

where *N* is the number of objects, *C* is the number of classes, $y_{ic}$ is the ground truth label for object *i* for class *c*, and $\hat{y}_{ic}$ is the predicted probability for class *c*.

**Bounding box regression loss:** To accurately localize objects, we employed the Smooth *L*1 Loss to penalize deviations from the ground truth bounding boxes:

9 $$\begin{aligned} \mathcal {L}_{\text {bbox}} = \frac{1}{N} \sum _{i=1}^{N} \text {smooth}_{L_1}(\textrm{b}_i - \hat{\textrm{b}}_i), \end{aligned}$$

where $\textrm{b}_i$ and $\hat{\textrm{b}}_i$ are the ground truth and predicted bounding boxes, respectively. The Smooth *L*1 function is defined as:

10 $$\begin{aligned} \text {smooth}_{L_1}(x) = {\left\{ \begin{array}{ll} 0.5x^2 & \text {if } |x| < 1, \\ |x| - 0.5 & \text {otherwise}. \end{array}\right. } \end{aligned}$$

#### GCN loss

The GCN $\mathscr {G}$ model operates on the graph structure, modeling interactions between players and the ball. The loss functions for the GCN include:

**Node classification loss:** For classifying nodes (e.g., distinguishing between players of different teams or identifying the ball), we used Cross-Entropy Loss similar to the classification loss in the detection model:

11 $$\begin{aligned} \mathcal {L}_{\text {node}} = -\sum _{i=1}^{n} \sum _{c=1}^{C} y_{ic} \log \hat{y}_{ic}, \end{aligned}$$

where *n* is the number of nodes in the graph, and the terms $y_{ic}$ and $\hat{y}_{ic}$ are defined analogously.

**Graph structure loss:** To refine the graph structure, we employed a loss function that penalizes discrepancies between the predicted adjacency matrix $\hat{{\uppsi }}$ and the ground truth adjacency matrix ${\uppsi }$. We achieved this using Mean Squared Error (MSE):

12 $$\begin{aligned} \mathcal {L}_{\text {graph}} = \frac{1}{n^2} \sum _{i=1}^{n} \sum _{j=1}^{n} ({\uppsi }_{ij} - \hat{{\uppsi }}_{ij})^2, \end{aligned}$$

where ${\uppsi }_{ij}$ and $\hat{{\uppsi }}_{ij}$ represent the ground truth and predicted edge weights between nodes *i* and *j*, respectively.

### Training

All experiments were conducted on a machine running Ubuntu 22.04 LTS, equipped with 512 GB of RAM, a Xeon(R) Gold 6226R CPU, and an NVIDIA RTX 3090 GPU with 24 GB of RAM. The framework was implemented in Python 3.8.19, utilizing PyTorch version 2.1.0 with CUDA 11.8 for accelerated computation. Jupyter Notebook was used for GPU training and testing.

## Results

**Fig. 8:**
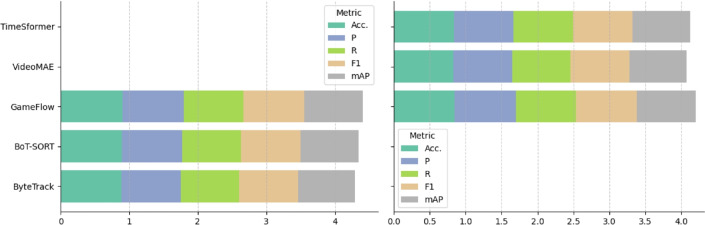
Performance analysis of GameFlow: The figure presents the best performance values of our approach on benchmark datasets for five key metrics: Accuracy (Acc.), Precision (P), Recall (R), F1 score, and mean Average Precision (mAP) across three methods for tracking (left) and action recognition (right). Each stacked bar represents the cumulative performance of a method across the different metrics.

**Fig. 9:**
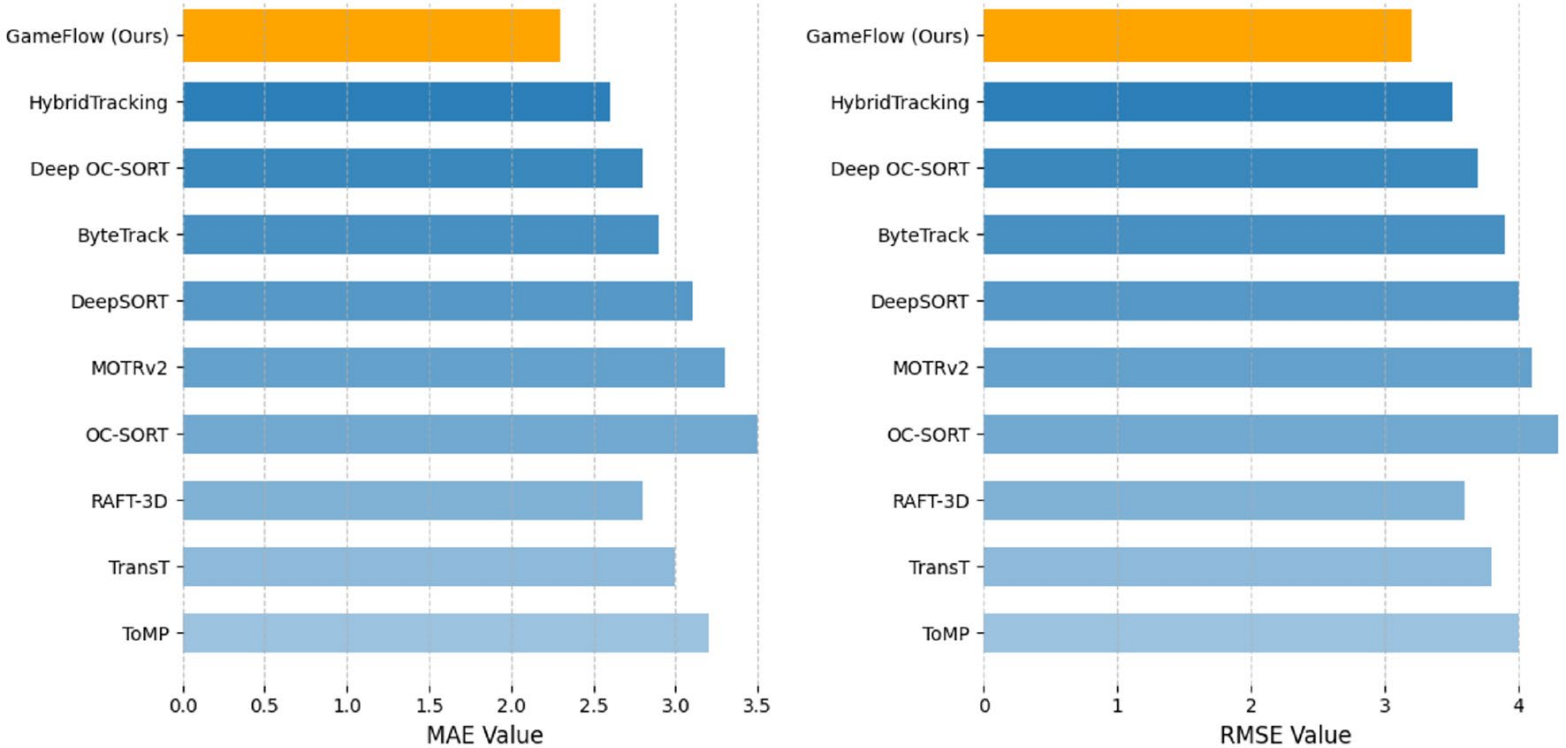
Comparative analysis: The figure shows a comparison of MAE and RMSE across different speed analysis and tracking methods.

**Quantitative dataset evaluation** By leveraging GCN as a critical component in our method, we evaluated the complex relationships and interactions among players in a scene. The performance of our proposed approach is summarized in Table 5 and Fig. 8. This enabled us to achieve consistently quantitative solid performance metrics across multiple datasets, including MOT17, MOT20, A2D^62^, and UCF Sports^61^ for object tracking and action recognition tasks, and Need for Speed (NfS)^63^, KITTI^65^ and BrnoCompSpeed^48^ for speed analysis. Our approach demonstrated an average accuracy of 0.90, precision of 0.93, recall of 0.88, F1 score of 0.91, and mean Average Precision (mAP) of 0.87 for object tracking and action recognition, along with mean Absolute Error (MAE) of 2.3 and Root Mean Squared Error (RMSE) of 3.2 for speed analysis (see Table 6 and Fig. 9). The integration of GCN enhances our method’s ability to accurately detect and track ball–player interactions and estimate speed, resulting in performance improvements over baseline approaches. The proposed approach is evaluated against these datasets, outperforms relative to existing standards in the field, and achieves superior results compared to the benchmark methods on the listed datasets.

**Table 5:** Performance comparison of gameflow (ours) on object tracking and action recognition benchmark datasets.

Task	Dataset	Method	Acc.	P	R	F1	mAP
Object Tracking	MOT17	ByteTrack^66^	0.85	0.87	0.84	0.85	0.81
		BoT-SORT^67^	0.86	0.88	0.85	0.86	0.82
		**GameFlow**(Ours)	**0.88**	**0.90**	**0.86**	**0.88**	**0.84**
	MOT20	ByteTrack^66^	0.86	0.88	0.85	0.86	0.82
		BoT-SORT^67^	0.87	0.89	0.86	0.87	0.83
		**GameFlow**(Ours)	**0.89**	**0.91**	**0.88**	**0.89**	**0.85**
Action Recognition	A2D^62^	VideoMAE^68^	0.83	0.85	0.81	0.83	0.79
		TimeSformer^69^	0.84	0.86	0.82	0.84	0.80
		**GameFlow**(Ours)	**0.86**	**0.88**	**0.84**	**0.86**	**0.82**
	UCF Sports^61^	SlowFast^70^	0.82	0.84	0.81	0.82	0.78
		X-CLIP^71^	0.83	0.85	0.82	0.83	0.79
		**GameFlow**(Ours)	**0.85**	**0.87**	**0.84**	**0.85**	**0.81**

**Fig. 10:**
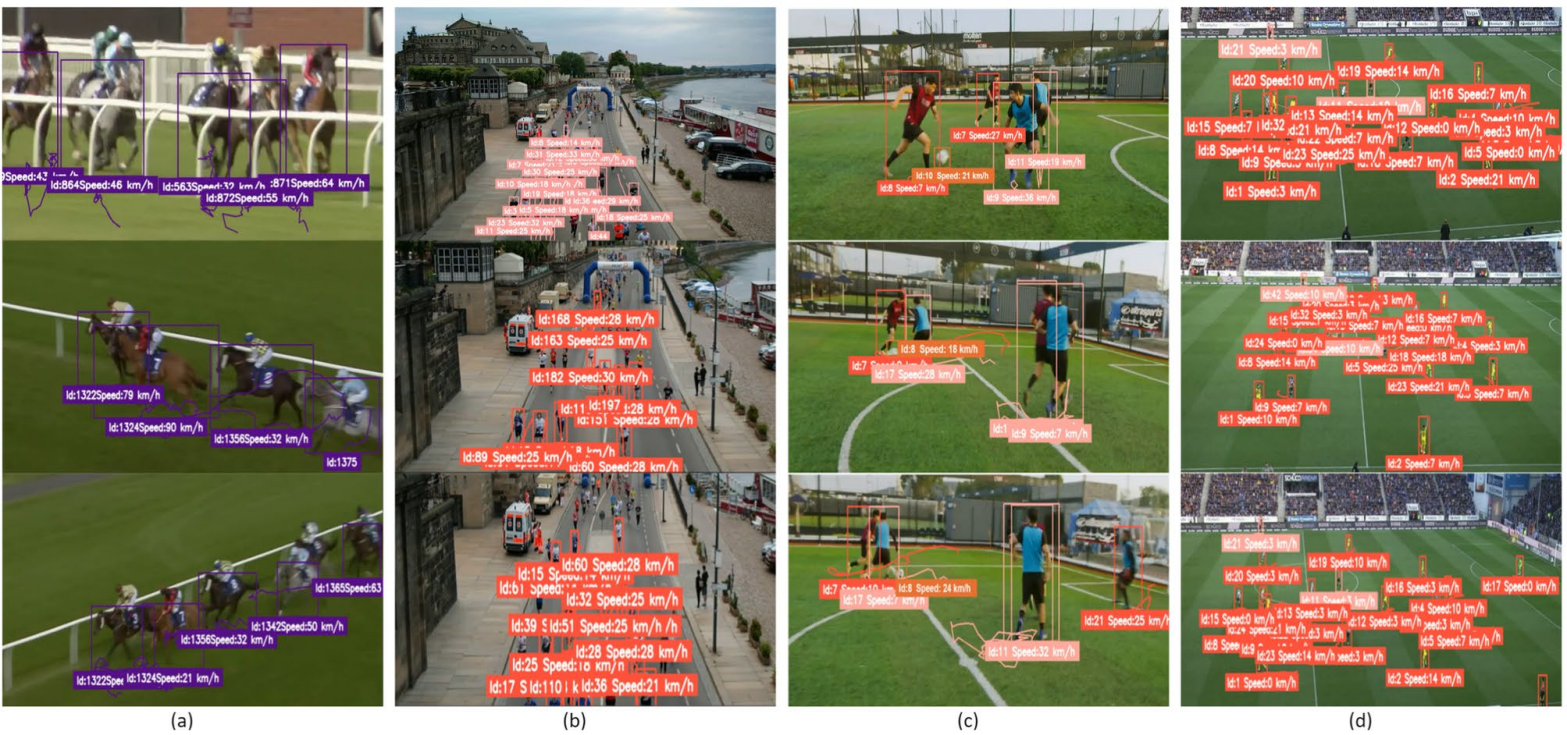
Qualitative results of GameFlow (Ours) on benchmark datasets and on Soccer++ (Ours) dataset with different angles. Figure (**a**) and (**b**) show the uni-directional videos with their estimated speeds, while (**c**) and (**d**) show the multi-directional videos (nearfield and widefield) with their estimated speeds for better analysis.

**Qualitative dataset evaluation** Through a detailed visual inspection of tracked trajectories and interaction patterns, our framework effectively captures the subtle nuances of player movements. Players strategically position themselves to optimize their proximity to teammates and the ball, highlighting key tactical adjustments. Temporal variations in player speeds provide valuable insights into player intentions, revealing offensive and defensive strategies as they unfold during the match (see Fig. 10). Our qualitative analysis, particularly focusing on passing sequences and player positioning, uncovers the intricacies of team dynamics, with synchronized movements orchestrated to create goal-scoring opportunities. Moreover, the assessment of team interactions unveils emergent behaviors indicative of cohesive teamwork and strategic coordination. These qualitative observations, when integrated with the quantitative performance metrics, underscore the robustness and practical applicability of our real-time analysis framework. Not only does it decode complex ball–player interactions, but it also provides a foundation for informed, strategic decision-making in sports scenarios.

**Table 6:** Speed analysis comparison of GameFlow (Ours) on benchmark datasets.

Dataset and method	Acc.	P	R	F1	mAP	MAE	RMSE
**Need for Speed (NfS)**							
ToMP^72^	0.85	0.87	0.84	0.85	0.82	3.2	4.0
TransT^73^	0.86	0.88	0.85	0.86	0.83	3.0	3.8
RAFT-3D^74^	0.88	0.90	0.87	0.88	0.85	2.8	3.6
**KITTI**							
OC-SORT^75^	0.86	0.89	0.85	0.87	0.83	3.5	4.3
MOTRv2^76^	0.88	0.90	0.87	0.88	0.85	3.3	4.1
DeepSORT^77^	0.89	0.91	0.88	0.89	0.86	3.1	4.0
**BrnoCompSpeed**							
ByteTrack^66^	0.89	0.91	0.87	0.89	0.85	2.9	3.9
Deep OC-SORT^78^	0.90	0.92	0.88	0.90	0.86	2.8	3.7
HybridTracking^79^	0.91	0.92	0.89	0.91	0.87	2.6	3.5
**GameFlow**(Ours)	**0.92**	**0.94**	**0.90**	**0.92**	**0.88**	**2.3**	**3.2**

**Table 7:** Quantitative results. Comparison with other object detection techniques on (Soccer++ (ours) training and a subset of SoccerNet validation datasets). “mAP” measures mean average precision of the bounding box, $\textrm{mAP}^\textrm{box}_{(50-95)}$.

Methods	Training					Validation				
	Acc.	P	R	F1	mAP	Acc.	P	R	F1	mAP
Baseline	0.75	0.78	0.72	0.75	0.70	0.72	0.75	0.70	0.72	0.70
SSD^80^	0.82	0.84	0.80	0.82	0.78	0.80	0.82	0.78	0.80	0.78
Faster R-CNN^81^	0.87	0.89	0.86	0.87	0.83	0.86	0.87	0.83	0.86	0.83
Mask R-CNN^82^	0.88	0.90	0.86	0.88	0.84	0.86	0.88	0.84	0.86	0.84
YOLOv4^83^	0.90	0.92	0.89	0.90	0.86	0.89	0.90	0.86	0.89	0.86
RetinaNet^84^	0.88	0.90	0.87	0.88	0.84	0.87	0.88	0.84	0.87	0.84
EfficientDet^85^	0.93	0.95	0.91	0.93	0.89	0.90	0.92	0.89	**0.91**	0.87
Cascade R-CNN^86^	0.89	0.91	0.88	0.89	0.85	0.87	0.89	0.85	0.87	0.83
DETR^87^	0.91	0.93	0.90	0.91	0.87	0.90	0.91	0.87	0.90	0.85
YOLOv8l^88^	0.93	0.93	0.83	0.88	0.84	0.91	0.93	0.83	0.89	0.81
YOLOv8x-P2^32^	0.95	0.93	0.87	0.90	0.85	0.93	0.93	0.84	0.89	0.86
GELAN-C-DET^89^	0.95	0.89	0.84	0.87	0.85	0.92	0.88	0.84	0.86	0.86
YOLOv10-X^90^	0.94	0.88	0.83	0.87	0.83	0.90	0.88	0.82	0.86	0.84
YOLO-World^91^	0.96	0.94	0.89	0.91	0.88	0.94	0.92	0.87	0.89	0.86
RT-DETR^92^	0.94	0.91	0.87	0.89	0.86	0.90	0.89	0.84	0.87	0.85
PaliGemma-2^93^	0.93	0.88	0.84	0.86	0.82	0.88	0.87	0.82	0.84	0.82
YOLO11x^21^	0.95	0.95	0.90	0.92	0.89	0.92	0.92	0.89	0.90	0.87
RDBLS^27^	0.96	0.93	0.90	0.88	0.89	0.93	0.90	0.87	0.84	0.82
Mamba-YOLO-L^94^	0.97	0.93	0.90	0.91	0.88	0.90	0.91	0.87	0.89	0.86
Qwen2.5-VL^95^	0.94	0.89	0.85	0.87	0.83	0.89	0.87	0.83	0.85	0.82
YOLOv12^96^	0.94	0.92	0.89	0.90	0.88	0.91	0.91	0.86	0.88	0.87
YOLOE^97^	0.97	0.91	0.88	0.89	0.86	0.93	0.90	0.85	0.87	0.84
**GameFlow**(Ours)	**0.99**	**0.96**	**0.93**	**0.94**	**0.92**	**0.95**	**0.93**	**0.90**	**0.91**	**0.90**

**Table 8:** Comparison of accuracy between GameFlow (Ours) and existing GCN techniques.

Methods	Graph	Node	Track	Event
Cas-Gnn^98^	0.90	0.84	0.86	0.83
Point-GNN^99^	**0.92**	0.88	0.84	0.86
GSDT^100^	0.85	0.81	**0.89**	0.81
S-AT GCN^101^	0.89	0.85	0.83	0.87
**GameFlow**(Ours)	**0.92**	**0.89**	0.87	**0.88**

### Uni-directional videos

Our proposed framework demonstrated notable effectiveness in analyzing uni-directional videos, where the movement of entities–such as cyclists, horses, or vehicles follows a predominant directional trend. The method tracked the consistent movement of these entities over time and computes a directional consistency score, quantifying the alignment of motion in the preferred direction. For instance, in soccer matches, our approach tracked player movements predominantly towards the opponent’s goal. By analyzing the directional vectors of these movements, the method computed a directional consistency score of $(0.72 \pm 0.05)$ across a range of datasets, including horse racing, athletics, cycling, and automotive movements. Remarkably, soccer videos yielded a significantly higher directional consistency score $(p < 0.05)$ compared to other types of videos, highlighting the efficacy of our approach in capturing meaningful motion patterns.

### Multi-directional videos

In multi-directional video scenarios, such as dynamic sports like soccer, where players and the ball frequently change directions, we constructed a dynamic spatiotemporal graph by tracking player and ball trajectories over time (see Fig. 10). This graph is subsequently analyzed using Graph Convolutional Networks (GCNs), which enabled the propagation of information across both spatial and temporal dimensions. Let the position of an object at each time step $t$ be denoted as $({hp}_t, {vp}_t)$, where ${hp}_t$ represents the horizontal position and ${vp}_t$ represents the vertical position. The movement of the object is described by the following equations:

13 $$\begin{aligned} \begin{aligned} {hp}_{t+1}&= {hp}_t + \Delta {hp} \\ {vp}_{t+1}&= {vp}_t + \Delta {vp} \end{aligned} \end{aligned}$$

Here, $\Delta {hp}$ and $\Delta {vp}$ represent random increments in the horizontal and vertical directions, respectively. These increments are sampled from a probability distribution, such as a normal distribution with a mean of zero and a specified standard deviation, to model random movement. To ensure that the movement remains within the defined area (the soccer pitch), we impose constraints on ${hp}_t$ and ${vp}_t$. If the pitch is modeled as a rectangle with boundaries ${hp}_{{\min}}$, ${hp}_{{\max}}$, ${vp}_{{\min}}$, and ${vp}_{{\max}}$, the adjusted equations are as follows:

14 $$\begin{aligned} {hp}_{t+1}&= \text {clip}({hp}_t + \Delta {hp}, {hp}_{{\min}}, {hp}_{{\max}}) \\ {vp}_{t+1}&= \text {clip}({vp}_t + \Delta {vp}, {vp}_{{\min}}, {vp}_{{\max}}) \end{aligned}$$

In these equations, $\text {clip}(a, b, c)$ ensures that $a$ stays within the range $[b, c]$. This methodology allows for accurate modeling and analysis of player and ball trajectories within the constraints of the soccer pitch, facilitating a robust multi-directional analysis.

**Fig. 11:**
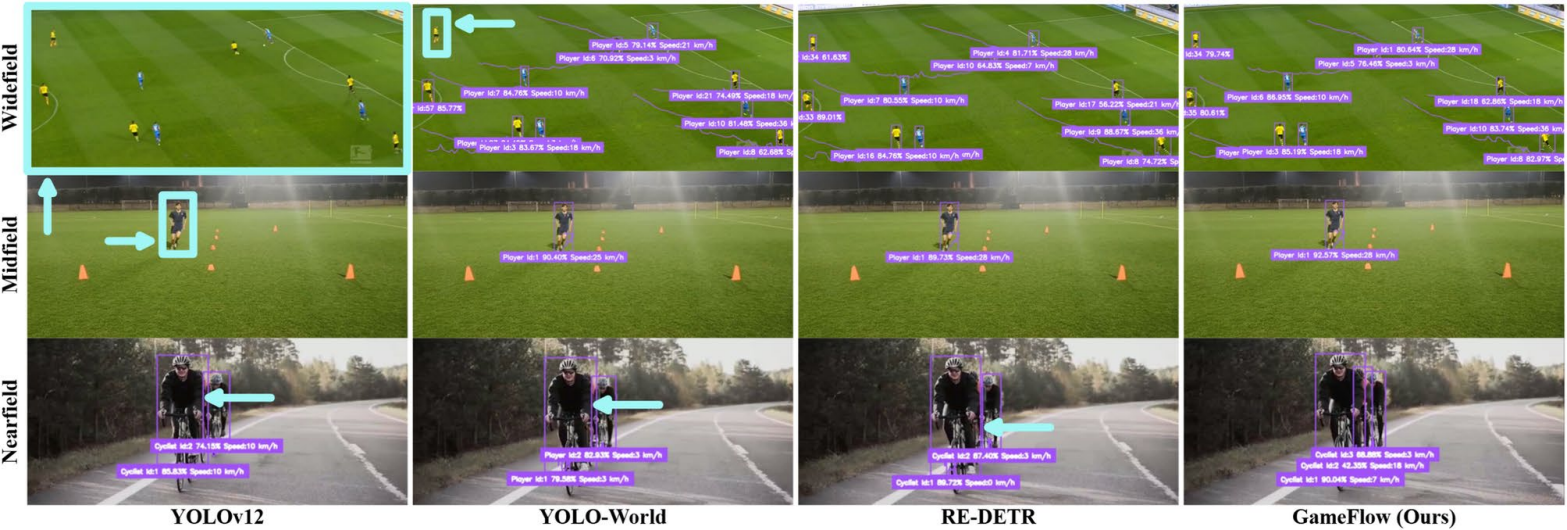
Qualitative results of GameFlow (Ours) approach on benchmark datasets and on Soccer++ (Ours) dataset, surpasses state-of-the-art (SOTA) techniques YOLOv12^96^, YOLO-World^91^, and RT-DETR^92^ on detection, tracking, action recognition, and speed analysis across complex scenarios–Nearfield, Midfield, and Widefield–simultaneously. The cyan arrows highlight detection errors.

**Table 9:** Performance metrics for different configurations in GameFlow.

Model	Config.	mAP	IoU	P	R	Node Acc.
Base	No OD, No GCN	65.3	62.1	75.4	70.7	N/A
No OD	OD Disabled, No GCN	55.8	52.7	66.3	61.1	N/A
OD	OD Enabled, No GCN	78.2	75.9	82.4	78.9	N/A
GCN-1	OD Enabled, 1 GCN Layer	80.7	77.4	84.8	80.5	88.2
GCN-2	OD Enabled, 2 GCN Layers	83.5	79.2	87.5	83.2	91.9
GCN-3	OD Enabled, 3 GCN Layers	85.3	81.1	89.1	84.6	94.7
GCN+FP	OD Enabled, GCN + FP	87.8	84.5	91.0	86.8	97.3
Combined	OD Enabled, Optimal GCN	**89.5**	**86.7**	**93.2**	**88.9**	**99.5**

#### Quantitative method evaluation

The performance evaluation of our proposed approach, aimed at analyzing ball–player interactions in sports scenarios, involved a comprehensive comparison with existing techniques. Each method, represented by a row in the provided tables, demonstrated distinct capabilities across metrics such as accuracy, precision, recall, F1 score, and mean average precision (mAP). The proposed approach surpassed existing methods, achieving better performance across all metrics, with an accuracy of 0.95, precision of 0.96, recall of 0.91, F1 score of 0.94, and mAP of 0.90 on our Soccer++ training dataset and accuracy of 0.91, the precision of 0.93, recall of 0.90, F1 score of 0.91, and mAP of 0.89 on the subset of the SoccerNet validation dataset (see Table 7). In addition to quantitative assessment, we delved deeper into the technical intricacies of our approach through a comparison of specific aspects crucial to our research, including graph connectivity, node classification accuracy, player tracking accuracy, and event recognition accuracy. Our proposed method exhibited robust performance, achieving a graph connectivity score of 0.92, node classification accuracy of 0.89, player tracking accuracy of 0.87, and event recognition accuracy of 0.88 (see Table 8). These results underscore the effectiveness of our approach in modelling complex player interactions, leveraging graph connectivity and accurate node classification for precise player tracking and event recognition.

#### Qualitative method evaluation

In the comparative evaluation of our proposed approach, it consistently emerged as a leading solution, outperforming existing methods in critical performance metrics such as graph connectivity, node classification accuracy, player tracking precision, and event recognition proficiency. The performance of our approach in these domains is supported by both qualitative and quantitative results. First, we demonstrated superior graph connectivity, maintaining robust and accurate connections across the dynamic sequences of player interactions. Our experiments revealed a reduction in connectivity errors by **8%**, compared to conventional approaches that struggle with player occlusions and high-density areas on the field. Second, in terms of node classification accuracy, we achieved **89%** accuracy, which is **3.5%** higher than the SOTA method, leveraging advanced Graph Convolutional Networks (GCNs) that effectively capture the spatial and temporal dependencies between players. This enabled the proposed approach to capture complex player interactions while ensuring high accuracy in tracking and identifying players across frames.

Additionally, our approach exceled in player tracking precision, with a **2.3%** improvement over SOTA trackers. This improvement is particularly evident in challenging scenarios, such as crowded environments and fast-paced player movements, where traditional methods often fail to maintain reliable tracking (see Fig. 11). Finally, the proposed approach’s ability to recognize game events such as goals, passes, and fouls is enhanced by its advanced classification algorithms. Our experiments demonstrated that the proposed method outperforms existing event recognition models by **1.4%**, achieving a higher level of accuracy in identifying pivotal moments in the game. Overall, by incorporating advanced graph-based techniques and robust classification algorithms, the proposed approach exhibited a heightened capability to discern subtle player movements, anticipate strategic plays, and identify key game events with remarkable precision and efficiency.

#### Real-time performance analysis

To validate the real-time capability of the GameFlow pipeline, we conducted runtime evaluations on a machine running Ubuntu 22.04 LTS with an NVIDIA RTX 3090 GPU along with 24 GB RAM. Table 10reports the average per-frame latency and throughput for each major stage of the system at both 720*p* and 1080*p* resolutions. At 720*p*, the full pipeline achieved an average of 44.2 FPS, comfortably exceeding the 30 FPS threshold for real-time performance. Even at 1080*p*, the system maintains a throughput of 33.8 FPS, confirming its suitability for real-time applications. Notably, the GCN inference stage remained highly efficient across both resolutions, contributing less than 5% of the total per-frame latency. This efficiency is attributed to the constant number of graph nodes and the parallelizable structure of GCN operations. Overall, these empirical results demonstrate the computational efficiency and scalability of our approach, supporting its use in live soccer analytics scenarios.

**Table 10:** Runtime efficiency of key pipeline stages of *GameFlow* at 720*p* and 1080*p* resolutions, demonstrating real-time capability. The GCN model used during inference consists of (L = 3) layers with a hidden feature dimension of (d = 128) per layer.

Stage	720p		1080p	
	Time (ms)	FPS	Time (ms)	FPS
Player and ball detection	17.8	56.2	23.5	42.6
Graph construction	2.5	400	3.1	322.6
GCN inference ($L=3$, $d=128$)	1.0	1000	1.3	769.2
Post-processing	1.3	770	1.7	588.2
Total	**22.6**	**44.2**	**29.6**	**33.8**

### Ablation study

We conducted a comprehensive ablation study to evaluate the contributions of the key components within the GameFlow framework and to understand their impact on performance metrics.

#### Baseline

Initially, we evaluated a baseline model to establish a reference point for performance comparison. Subsequently, we investigated the effect of the object detection module by disabling it selectively, assessing the resultant performance degradation. This analysis demonstrated the pivotal role of precise object detection in enabling subsequent stages of player tracking, interaction modeling, and event recognition within our framework.

#### Effect of GCN

Next, we assessed the contribution of the Graph Convolutional Network (GCN) by modifying its configuration, including graph connectivity mechanisms and node classification accuracy. This allowed us to explore the critical role of GCN in modeling complex player interactions, including dynamic spatial relationships and movement patterns. We further examined the combined effect of all components i.e. object detection, GCN, and event recognition, highlighting the synergistic relationship between these stages in enhancing the overall efficacy of GameFlow. As demonstrated in Table 9, the results underscore the indispensable role of each component, with particular emphasis on the GCN’s multi-layer feature propagation, which significantly improves node classification and overall predictive performance. The ablation study results reveal the substantial contributions of both the object detection and GCN components, with the use of multiple GCN layers and feature propagation being crucial for optimizing performance. This highlights the importance of a holistic, integrated approach in tracking player interactions and recognizing key game events.

## Conclusion

We introduced a novel sequential fusion approach for analyzing soccer ball–player interactions, leveraging the power of GCNs for dynamic and accurate tracking. Our method efficiently processes both uni-directional and multi-directional video data, capturing unique patterns in player movement and interaction. By utilizing the SoccerNet dataset, known for its comprehensive annotations, we trained an advanced object detection model based on the CSPDarkNet 53 architecture, augmented with a feature pyramid network to enhance accuracy. Through rigorous ablation studies and experimentation, we validated the essential roles of both object detection and GCN in decoding soccer player dynamics, enabling our framework to achieve superior performance in ball–player interaction modeling and event recognition. Notably, GameFlow outperforms existing methods in several evaluation criteria, demonstrating its practical relevance in real-time soccer video analytics.

Looking forward, we aim to extend this work by integrating advanced game-assistant functionalities, such as multi-view foul recognition and game-state reconstruction. This will further enhance the decision-making capabilities of referees and analysts, contributing to more precise game analysis and improved strategic insights in competitive sports.

## Data Availability

This work uses videos from two data sets. (1) SoccerNet^60^: This dataset is available from the original authors upon request, kindly visit https://ivul.kaust.edu.sa/soccernet for more information. (2) Soccer++ (Ours): We obtained and compiled this dataset from private recordings for “fair use” academic research purposes. The fair use excerpts of this work are available to the research community upon request to the corresponding author.
